# Cateterismo de artéria mesentérica para tratamento de trombose de veia porta

**DOI:** 10.1590/1677-5449.008416

**Published:** 2017

**Authors:** Guilherme Benjamin Brandão Pitta, Deise Azevedo Pereira, Milena de Fátima Queiroz Oliveira, Eduardo Abadie Guedes, Joaquim Araújo Sampaio

**Affiliations:** 1 Universidade Estadual de Ciências da Saúde de Alagoas – UNCISAL, Faculdade de Medicina, Maceió, AL, Brasil.; 2 Universidade Federal de Alagoas – UFAL, Faculdade de Medicina, Maceió, AL, Brasil.; 3 Hospital Memorial Arthur Ramos – HMAR, Cirurgia Vascular, Maceió, AL, Brasil.

**Keywords:** veia porta, trombose, trombofilia, isquemia mesentérica, abdome agudo

## Abstract

A trombose de veia porta é uma causa rara de abdome agudo vascular e está diretamente relacionada a trombofilias hereditárias ou adquiridas. O caso de um paciente de 60 anos, sexo masculino, com quadro clínico de isquemia mesentérica confirmada por exame de imagem é apresentado. Foi submetido a enterectomia e enteroanastomose e, após esplenoportografia que detectou trombose de veia porta, indicou-se tratamento medicamentoso com infusão contínua de ativador tecidual do plasminogênio recombinante (Alteplase) através de cateterismo seletivo da artéria mesentérica superior. Trata-se de um tratamento inovador. Obteve-se sucesso na recanalização do sistema porta. O paciente evoluiu com quadro de sepse abdominal, necessitando de assistência em terapia intensiva por 25 dias. Evoluiu bem e recebeu alta hospitalar com o uso de anticoagulante. O artigo apresenta uma breve revisão de literatura e discussão do caso clínico.

## INTRODUÇÃO

A trombose de veia porta (TVPo) é um evento pouco comum nos pacientes não cirróticos e não neoplásicos. Aproximadamente 60% dos casos estão associados a condições trombofílicas, em especial doenças mieloproliferativas e trombofilias hereditárias^1^^-^ 3. A proporção de doentes com TVPo idiopática vem diminuindo com o diagnóstico recente de mais fatores de risco trombóticos hereditários^4^. Apesar de rara, a TVPo é potencialmente fatal quando complicada por isquemia intestinal^5^.

A apresentação clínica da TVPo varia desde um quadro assintomático, em obstruções parciais, até insuficiência hepática e óbito, em casos agudos^6^. A esplenoportografia (EPG) e a tomografia computadorizada (TC) de abdome com contraste são usadas de forma segura para o diagnóstico dessa doença, cujo tratamento é individualizado e pode ser realizado através de anticoagulação, terapia trombolítica sistêmica ou guiada por cateter, e terapia cirúrgica, quando há evolução do paciente para necrose intestinal^1^^,^ 7 ^,^ 8.

Este trabalho objetiva relatar o caso de um paciente de 60 anos com TVPo por trombofilia que evoluiu para necrose intestinal e realizar uma breve revisão de literatura sobre a doença.

## PARTE I: SITUAÇÃO CLÍNICA

Homem, 60 anos, natural e residente em Maceió (AL), foi admitido na emergência queixando-se de dor abdominal difusa havia 48 horas, de intensidade crescente, acompanhada de náuseas e vômitos. Relatou tratamento prévio para trombose venosa profunda (TVP) em membro inferior esquerdo. Era hipertenso, em uso irregular de losartana potássica, dislipidêmico e portador de esteatose hepática, além de ex-tabagista, etilista social e sedentário. Com relação aos antecedentes familiares, três irmãs e uma filha apresentaram TVP.

Durante o exame físico, estava consciente, agitado, sudoreico, taquipneico, afebril, acianótico, anictérico, hipertenso (150×80 mmHg), com mucosas hipocoradas (++/4+) e hidratadas. O abdome era globoso, distendido, tenso e doloroso à palpação profunda, com maior intensidade em fossa ilíaca direita e ruídos hidroaéreos diminuídos globalmente. Apresentava extremidades perfundidas, pulsos periféricos palpáveis e amplos, sem edemas em membros inferiores e com panturrilhas livres.

**Figura 1:**
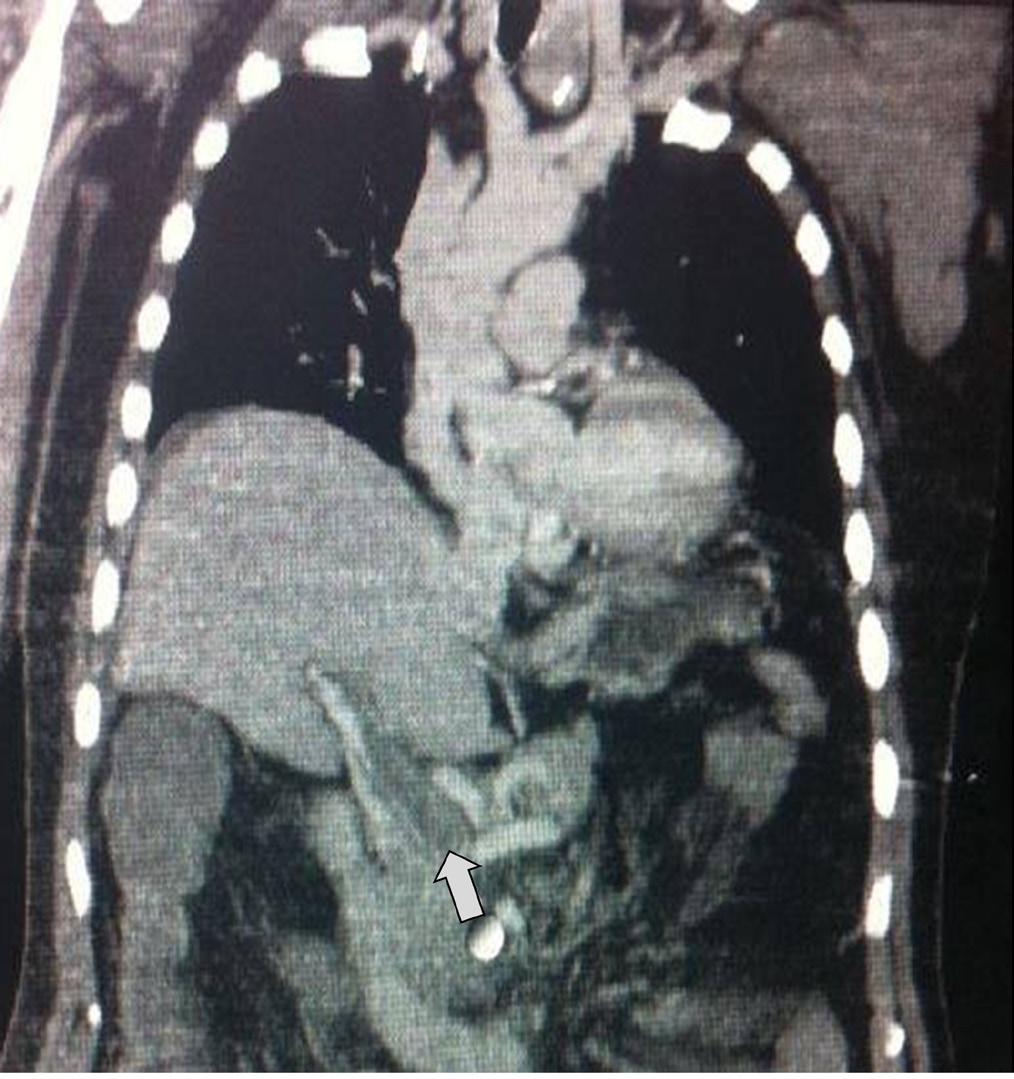
Tomografia computadorizada de tórax e abdome evidenciando trombose em veia porta (indicada pela seta).

**Figura 2:**
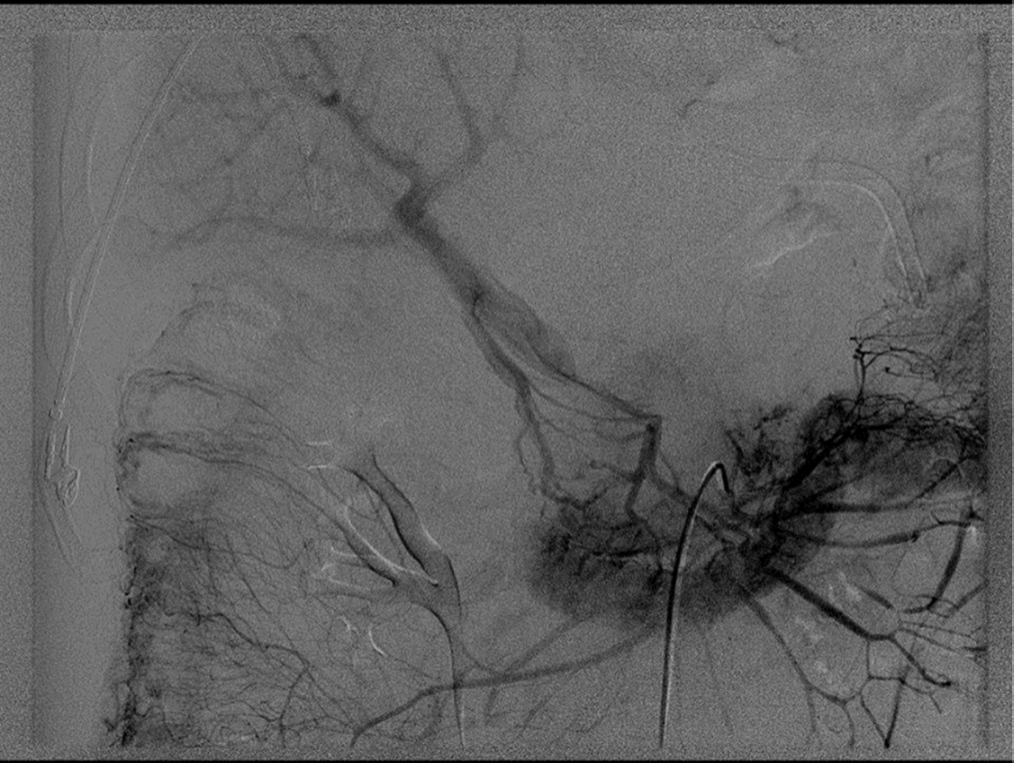
Esplenoportografia (fase venosa) evidenciando ausência de retorno venoso pelas veias mesentérica superior e esplênica, com drenagem pelas veias colaterais.

Hemograma e dosagens bioquímicas revelaram discreta leucocitose (13.100/mm^3^) acompanhada de neutrofilia (10.083/mm^3^) e hiperglicemia (146 mg/dL). As demais dosagens bioquímicas encontravam-se dentro da normalidade. Foi realizada TC de tórax e de abdome com contraste, que detectou TVPo (Figura 1), sendo encaminhado imediatamente aos serviços da cirurgia vascular. A EPG evidenciou ausência de retorno venoso pela veia mesentérica superior e veia esplênica, tendo como local de oclusão a origem da veia porta (Figura 2).

## PARTE II: O QUE FOI FEITO

**Figura 3:**
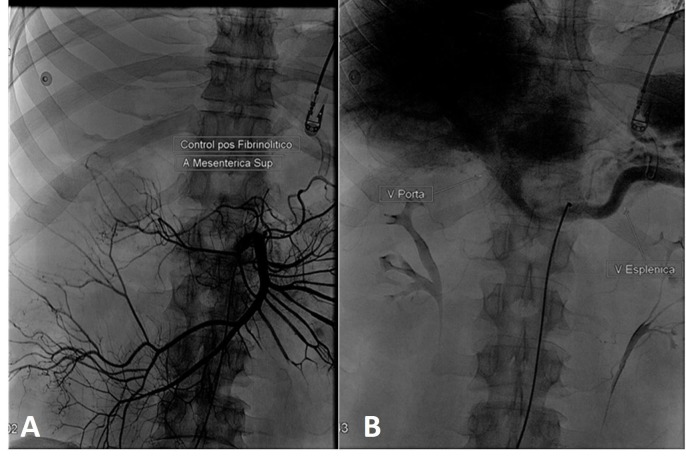
Esplenoportografia de controle pós-trombólise por cateterismo da artéria mesentérica superior (3A), evidenciando veia porta (à esquerda) e veia esplênica (à direita) pérvias (3B).

A conduta inicial foi a realização de laparotomia exploratória devido ao quadro de abdome agudo, evidenciando extensa área necrótica de jejuno-íleo, com indicação de enterectomia com enteroanastomose, procedimento que removeu aproximadamente 60% do segmento jejunal do paciente. No dia seguinte, iniciou-se a recanalização do sistema porta por trombólise através de cateterismo seletivo de artéria mesentérica superior, com o ativador tecidual do plasminogênio recombinante (Alteplase), 10 mg em *bolus* e 40 mg a cada 24 horas por 3 dias, seguido de heparinização plena em bomba de infusão contínua. A EPG de controle no quarto dia de internamento (Figura 3) evidenciou retorno do fluxo sanguíneo em veia porta e melhora de perfusão hepática.

O seguimento pós-operatório foi realizado em UTI, onde o paciente permaneceu grave, sedado, sob ventilação mecânica, hemodinamicamente instável e em uso de droga vasoativa por 6 dias. Evoluiu para sepse grave. Foi transferido para um hospital em São Paulo, onde permaneceu em UTI por 19 dias e em uso de heparina de baixo peso molecular. Desenvolveu trombocitopenia induzida por heparina e, devido à demora no diagnóstico, complicou com novo episódio de TVP. Substituiu-se a heparina por fondaparinux, com normalização do número de plaquetas e melhora do quadro. Permaneceu internado em enfermaria por mais 20 dias, recebendo alta hospitalar com o uso de anticoagulante oral.

Durante investigação posterior, encontrou-se na família mutação da metilenotetrahidrofolato redutase, porém sem aumento da homocisteína. Também foi observado polimorfismo do gene inibidor do ativador do plasminogênio 1 (PAI-1), mas não se chegou a conclusão diagnóstica. Demais exames foram normais ou negativos, como mutação do fator V de Leiden, da antitrombina, proteínas S e C, anticardiolipina e anticoagulante lúpico.

## DISCUSSÃO

A isquemia mesentérica por TVPo com infarto intestinal é uma complicação grave e temida, associada a uma mortalidade de 60%^9^, necessitando de abordagem cirúrgica com possibilidade de extensa ressecção intestinal. Sinais de peritonite constituem indicação de laparotomia exploradora e ressecção das áreas necróticas^8^^,^ 10 ^-^ 12. Evoluções para óbito ocorrem em 20-50% dos casos de infarto intestinal^1^^,^ 13 ^,^ 14. A TVPo é um caso raro e importante de abdome agudo vascular.

A confluência das veias esplênica e mesentérica superior, posteriormente ao colo do pâncreas, origina a veia porta, que drena o sangue proveniente do trato gastrointestinal abdominal e pâncreas para o fígado^5^. A interrupção desse fluxo promove o aparecimento de mecanismos compensatórios, que incluem a vasodilatação reflexa da artéria hepática e a formação de vasos colaterais, permitindo que o sangue contorne o local da obstrução^15^^,^ 16.

Quanto à etiologia, a TVPo classificada em não maligna e não cirrótica^17^ inclui malformação vascular e estados de hipercoagulabilidade^1^, como deficiência de antitrombina III, proteínas C e S, disfibrinogenemia e mutação do G20210A do gene da protrombina^1^^,^ 5 ^,^ 17 ^,^ 18. Cerca de 60% dos pacientes com trombose mesentérica apresentam passado de TVP^5^^,^ 7. O paciente do caso apresentava história pessoal e familiar de TVP. Suspeitou-se de uma TVPo de etiologia hereditária. Foram encontrados mutação da metilenotetrahidrofolatoredutase e polimorfismo do gene do PAI-1, sem conclusão sobre aumento de estado de hipercoagulabilidade, devido a valores normais de homocisteína. O número de casos de TVPo verdadeiramente “idiopática” reduziu devido à identificação de causa subjacente em 80% dos pacientes rigorosamente investigados^14^.

O quadro clínico da TVPo envolve complicações relacionadas à hipertensão portal em 30% dos casos^1^, como ascite, surgimento de varizes gástricas e esofágicas, e hemorragia digestiva alta^1^^,^ 7. A trombose de veia mesentérica é responsável por 5-15% dos quadros de isquemia mesentérica^7^^,^ 13 ^,^ 19. Inicialmente, há isquemia da mucosa, que, ao evoluir para infarto transmural, ocasiona peritonite difusa^10^.

A ultrassonografia é considerada a primeira linha para diagnóstico de desordens no sistema venoso portal, mas não foi utilizada devido ao quadro de abdome agudo. Apresenta especificidade e sensibilidade acima de 80%, que aumentam com o uso do Doppler^2^^,^ 20. Presença de material ecogênico aderido à parede do vaso determinando obstrução parcial da luz, colaterais venosas portais, aumento do calibre da veia porta e transformação cavernomatosa, ausência de fluxo no vaso ao Doppler e fluxo arterial de alta frequência devido à vasodilatação da artéria hepática são achados comuns^20^. A TC contrastada de abdome total ou a ressonância magnética com contraste podem ser utilizadas, cujos achados são: falha no enchimento da veia porta ou aumento da luz da veia^2^. Por ser mais rápida e menos incômoda ao paciente, o exame de escolha na presença de abdome agudo é a TC contrastada de abdome^2^.

A EPG possibilita melhor visualização do tamanho do trombo, localização e acometimento do fluxo sanguíneo, apresentando sensibilidade diagnóstica de 90%^19^. O procedimento envolve duas fases: primeiro a fase de injeção de contraste na artéria mesentérica superior, com visualização do território arterial, e depois a fase venosa, na qual obstrução venosa e presença de trombos intraluminais são registradas^11^. No caso descrito, a obstrução localizava-se na confluência das veias esplênica e mesentérica superior (Figura 2), cujo acometimento determinou isquemia mesentérica, que é a principal complicação da TVPo aguda^4^.

O tratamento da TVPo aguda é individualizado e depende da causa da trombose^12^. A Associação Americana para o Estudo das Doenças do Fígado recomenda, nos quadros agudos de pacientes não cirróticos e sem malignidade, heparinização plena por 2 a 3 semanas como terapia inicial, seguida de inibidores da vitamina K, de modo a manter a razão normalizada internacional (RNI) entre 2 e 3. Antes do início da anticoagulação, os pacientes devem ser avaliados quanto à presença de hipertensão portal, varizes esofágicas ou trombocitopenia devido ao hiperesplenismo, a fim de avaliar o risco de complicações hemorrágicas^1^^,^ 12. Cerca de 20% dos pacientes trombofílicos apresentaram recorrência da trombose^21^.

Quando há falha da terapia de anticoagulação ou acometimento da veia mesentérica superior, pode-se considerar o tratamento sistêmico com o uso de trombolítico associado a heparina de baixo peso molecular. Quando há contraindicação à anticoagulação sistêmica, a trombólise guiada por cateter é indicada^12^^,^ 22, cujo acesso pode ser direto (transjugular, trans-hepático e transesplênico) ou indireto, injetando-se trombolítico na artéria mesentérica superior^22^. Trombectomia cirúrgica é proscrita com alto índice de recorrência^3^. No caso descrito, a conduta escolhida foi trombólise guiada por cateter posicionado em artéria mesentérica superior, associada a heparinização plena. Evidência crescente apoia o uso da terapia trombolítica precoce em pacientes com TVPo aguda^14^. Altas taxas de recanalização foram vistas com trombólise em comparação ao tratamento conservador com anticoagulação^14^.

A TVPo, quando complicada com infarto intestinal, apresenta altos índices de morbimortalidade^14^. Devido ao quadro de abdome agudo, a ressecção é emergencial, e o tratamento da causa base evita o surgimento de novas áreas necróticas.

Não há estudos sobre quando a terapia trombolítica deve ser preferida à anticoagulação, mas há demonstração da eficácia da primeira quando a terapia com heparina não obteve sucesso, ficando reservada aos pacientes com TVPo grave e sem resposta à anticoagulação^1^. Devido à trombose extensa e grave, optou-se pela combinação de heparinização sistêmica e trombólise guiada por cateter com sucesso no reestabelecimento da circulação portal. Um possível transtorno protrombótico subjacente justificou o uso contínuo de anticoagulante oral^14^^,^ 23.
