# Supplementary material for: Cateterismo de artéria mesentérica para tratamento de trombose de veia porta
Source: J Vasc Bras. 2017 Jan-Mar;16(1):43–7. [Article in Portuguese] doi: 10.1590/1677-5449.008416 (PMC5829691; doi:10.1590/1677-5449.008416)
Supplement: Translation

# Translation: Catheterization of the mesenteric artery to treat portal vein thrombosis

**Authors:** Guilherme Benjamin Brandão Pitta, Deise Azevedo Pereira, Milena de Fátima Queiroz Oliveira, Eduardo Abadie Guedes, Joaquim Araújo Sampaio

**Affiliations:** 1 Universidade Estadual de Ciências da Saúde de Alagoas – UNCISAL, Faculdade de Medicina, Maceió, AL, Brazil.; 2 Universidade Federal de Alagoas – UFAL, Faculdade de Medicina, Maceió, AL, Brazil.; 3 Hospital Memorial Arthur Ramos – HMAR, Cirurgia Vascular, Maceió, AL, Brazil.

**Keywords:** portal vein, thrombosis, thrombophilia, mesenteric ischemia, acute abdomen

## Abstract

Portal vein thrombosis is a rare vascular cause of acute abdomen and it is directly related to hereditary or acquired thrombophilias. This article presents the case of a 60-year-old male patient, with clinical signs of mesenteric ischemia that was confirmed by imaging examination. He underwent enterectomy and enteroanastomosis and, after detection of portal vein thrombosis by splenoportography, he was prescribed drug-based treatment with continuous infusion of recombinant tissue plasminogen activator (Alteplase) via selective catheterization of the superior mesenteric artery. This is a treatment innovation. The portal system was successfully recanalized. However, the patient developed abdominal sepsis and required intensive care for 25 days. His clinical status improved and he was discharged with a prescription for oral anticoagulant. This article presents a brief review of the literature and a discussion of portal vein thrombosis.

## INTRODUCTION

Portal vein thrombosis (PVT) is an uncommon event among patients who do not have cirrhosis or cancer. Approximately 60% of cases are associated with prothrombotic conditions, especially myeloproliferative diseases and hereditary thrombophilias.^1^^-^ 3 The proportion of cases defined as idiopathic PVT has reduced after recent identification of additional hereditary thrombotic risk factors.^4^ Although rare, PVT is potentially fatal when complicated by intestinal ischemia.^5^

The clinical presentation of PVT ranges from asymptomatic cases, in which obstructions are partial, to liver failure and death, in acute cases.^6^ Splenoportography (SPG) and abdominal computed tomography (CT) with contrast are safe methods for diagnosing this disease, while treatment is individualized and may involve anticoagulation, systemic or catheter-guided thrombolysis, and/or surgery, in cases that progress to intestinal necrosis.^1^^,^ 7 ^,^ 8

This article describes the case of a 60-year-old patient with PVT caused by thrombophilia that progressed to intestinal necrosis and presents a brief review of the literature on this disease.

## PART I: CLINICAL CASE

A 60-year-old male patient, born and living in Maceió, AL, Brazil, was admitted to the emergency department complaining of diffuse abdominal pains with onset 48 hours previously and of increasing intensity, accompanied by nausea and vomiting. He reported having been treated previously for deep venous thrombosis (DVT) in the left lower limb. He was hypertensive, an irregular user of losartan potassium, and had dyslipidemia and hepatic steatosis, in addition to being an ex-smoker, a social drinker and sedentary. His family history included three sisters and a daughter who had had DVTs.

During the physical examination, he was conscience and agitated, with sudoresis and tachypnea, but without fever, cyanosis, or jaundice, and had hypertension (150×80 mmHg), with pallid (++/4+) and hydrated mucosas. His abdomen was swollen, distended, and tense, while deep palpation provoked pain, of greater intensity at the right iliac fossa, and bowel sounds were reduced in all areas. Extremities were perfused, peripheral pulses were palpable and robust, and he was free from lower limb edema and his calves were normal.

Blood tests and biochemical assays revealed discrete leukocytosis (13,100/mm^3^) and neutrophilia (10,083/mm^3^) and hyperglycemia (146 mg/dL). All other biochemical assay results were within normal limits. A CT of the thorax and abdomen with contrast detected PVT (Figure 1) and the patient was immediately transported to the vascular surgery service. An SPG reveled that there was no venous return via the superior mesenteric and splenic veins and showed that the site of occlusion was the origin of the portal vein (Figure 2).

**Figure 1:** Computed tomography of chest and abdomen, showing thrombosis in the portal vein (arrowed).

**Figure 2:** Splenoportography (venous phase) showing absence of venous return via the superior mesenteric and splenic veins, with drainage via collateral veins.

## PART II: WHAT WAS DONE

Initial conduct was an exploratory laparotomy to investigate the cause of acute abdomen, which found an extensive area of jejunum-ileum necrosis, with indication for enterectomy and enteroanastomosis. During the procedure, approximately 60% of the patient’s jejunal segment was removed. On the next day, recanalization of the portal system by thrombolysis was initiated with selective catheterization of the superior mesenteric artery and administration of recombinant tissue plasminogen activator (Alteplase), 10 mg in bolus and 40 mg every 24 hours for 3 days, followed by full heparinization via continuous infusion pump. A control SPG on the fourth day after admission (Figure 3) showed that blood flow through the portal vein had been restored and hepatic perfusion had improved.

**Figure 3:** Control splenoportography after thrombolysis by superior mesenteric artery catheterization (3A), showing patent portal vein (left) and splenic vein (right) (3B).

Postoperative follow-up was provided in the ICU, since the patient remained critical, sedated, on mechanical ventilation, hemodynamically unstable, and on vasoactive drugs for 6 days. He developed severe sepsis and was transferred to a hospital in São Paulo, where he remained in the ICU for a further 19 days, on low molecular weight heparin. He developed heparin-induced thrombocytopenia and, because of a delay in diagnosis, this was complicated by a further DVT episode. Heparin was substituted with fondaparinux and his platelet count and general condition improved. He remained in a standard ward for another 20 days and then he was discharged from hospital on oral anticoagulant.

During later investigations, it was found that there was a methylenetetrahydrofolate reductase mutation in the family, although homocysteine was not increased. A polymorphism of the plasminogen activator inhibitor 1 (PAI-1) gene was also detected, but no diagnostic conclusions could be made. Other test results were normal or negative, such as mutation of factor V Leiden, antithrombin, S and C proteins, anticardiolipin, and lupus anticoagulant.

## DISCUSSION

Mesenteric ischemia due to PVT with intestinal infarction is a severe and greatly feared complication that is associated with a mortality rate of 60%^9^ and requires surgical management that may involve extensive intestinal resection. Signs of peritonitis indicate a need for exploratory laparotomy and resection of necrotic areas.^8^^,^ 10 ^-^ 12 From 20 to 50% of intestinal infarction cases result in death.^1^^,^ 13 ^,^ 14 Portal vein thrombosis is a rare but important cause of vascular acute abdomen.

The confluence of the splenic and superior mesenteric veins posterior to the neck of the pancreas gives rise to the portal vein, which drains blood from the abdominal gastrointestinal tract and pancreas to the liver.^5^ Compensatory mechanisms are activated in response to interruption of this flow, including reflexive vasodilation of the hepatic artery and formation of collateral vessels, allowing blood to bypass the site of obstruction.^15^^,^ 16

Etiologies of PVT other than those related to cancer and cirrhosis^17^ include vascular malformations and hypercoagulable states,^1^ such as deficiencies of antithrombin III or deficiencies of protein C and protein S, dysfibrinogenemia, and the G20210A prothrombin gene mutation.^1^^,^ 5 ^,^ 17 ^,^ 18 Around 60% of patients with mesenteric thrombosis have a history of DVT.^5^^,^ 7 The patient in the case described here had both personal and family history of DVT. This raised the suspicion of PVT with a hereditary etiology. Tests revealed a methylenetetrahydrofolate reductase mutation and polymorphism of the PAI-1 gene, but no conclusive diagnosis of an increased hypercoagulable state could be made, since homocysteine levels were normal. The number of cases of PVT that are considered truly “idiopathic” has been reduced by identification of underlying causes in 80% of patients by rigorous investigation.^14^

The clinical presentation of PVT cases involves complications related to portal hypertension in 30% of cases,^1^ including ascites, appearance of gastric and esophageal varices, and upper digestive hemorrhage.^1^^,^ 7 Thrombosis of the mesenteric vein is responsible for 5-15% of cases of mesenteric ischemia.^7^^,^ 13 ^,^ 19 Initially, there is ischemia of the mucosa, which causes diffuse peritonitis as it progresses to transmural infarction.^10^

Ultrasonography is considered the first line for diagnosis of disorders in the portal vein system, although it was not used in this case because of the acute abdomen presentation. It offers specificity and sensitivity greater than 80%, which improve if Doppler imaging is employed.^2^^,^ 20 Common findings include, echogenic material adhering to the wall of the vessel causing partial obstruction of the lumen, collateral portal veins, increases in portal vein caliber and cavernous transformation, absence of flow through the vessel on Doppler, and high frequency arterial flow caused by vasodilation of the hepatic artery.^20^ Full abdominal CT with contrast or magnetic resonance with contrast can also be used and the following findings are relevant: failure of portal vein filling or increased vein lumen.^2^ Since it is quicker and more comfortable for the patient, the examination of choice for acute abdomen is abdominal CT with contrast.^2^

Splenoportography provides better images of size of thrombus, site, and compromised blood flow, offering diagnostic sensitivity of 90%.^19^ The procedure is conducted in two phases: first contrast is injected into the superior mesenteric artery and the arterial territory is examined, then a venous phase is conducted and any venous obstruction and intraluminal thrombi are recorded.^11^ In the case described here, the obstruction was located at the confluence of the splenic and superior mesenteric veins (Figure 2) and it was this involvement that caused mesenteric ischemia, which is the principal complication of acute PVT.^4^

Treatment of acute PVT is on a case-by-case basis and is dependent on the cause of the thrombosis.^12^ For acute cases in patients free from cirrhosis and cancer, the American Association for the Study of Liver Diseases recommends full heparinization for 2 to 3 weeks as initial treatment, followed by vitamin K inhibitors to maintain a international normalized ratio of between 2 and 3. Before starting anticoagulation, patients should be assessed for portal hypertension, esophageal varices, and thrombocytopenia due to hypersplenism, in order to evaluate the risk of hemorrhagic complications.^1^^,^ 12 In one study, around 20% of prothrombotic patients had a recurrence of thrombosis.^21^

When anticoagulation fails or the superior mesenteric vein is involved, systemic treatment with a thrombolytic combined with low molecular weight heparin should be considered. In cases in which systemic anticoagulation is contraindicated, guided thrombolysis by catheter is indicated,^12^^,^ 22 either via direct access (transjugular, trans-hepatic, or trans-splenic) or indirectly, injecting a thrombolytic agent into the superior mesenteric artery.^22^ Surgical thrombectomy is contraindicated and is associated with a high rate of recurrence.^3^ In the case described here, the conduct chosen was guided thrombolysis with a catheter positioned in the superior mesenteric artery, combined with full heparinization. There is growing evidence to support the use of early thrombolytic treatment for patients with acute PVT.^14^ High rates of recanalization have been observed with thrombolysis, when compared with conservative treatment with anticoagulation.^14^

When PVT is complicated by intestinal infarction, morbidity and mortality rates are high.^14^ When presentation is with acute abdomen, resection is conducted as an emergency procedure and treatment of the underlying cause prevents new areas of necrosis from appearing.

There are no studies of when thrombolysis should be preferred to anticoagulation, but it has been shown that the first of these offers efficacy when treatment with heparin is unsuccessful, and so it is reserved for patients with severe PVT who fail to respond to anticoagulation.^1^ In the case described here, because of the extensive and severe thrombosis, it was decided to employ a combination of systemic heparinization and guided thrombolysis via catheter, which successfully reestablished circulation through the portal vein. A possible underlying prothrombotic disorder justified continuous oral anticoagulation.^14^^,^ 23
